# Bayesian Analysis of Dietary Diversity among Lactating Mothers in Finote Selam District, Northwest Ethiopia: A Cross-Sectional Study

**DOI:** 10.1155/2021/9604394

**Published:** 2021-08-29

**Authors:** Yenesew Fentahun Gebrie, Tadesse Mihretie Dessie

**Affiliations:** ^1^Department of Statistics, Debre Markos University, Debre Markos, Ethiopia; ^2^Department of Applied Human Nutrition, Marie Stopes Ethiopia, Ethiopia

## Abstract

**Background:**

Dietary diversity is an essential element of diet quality. Lactation is one of the most complex and nutritionally demanding phases of the human life cycle, and the breastfed infant is dependent on mother nutrition. The objective of this study was to assess the prevalence of dietary diversity and its predictors among lactating mothers.

**Methods:**

A cross-sectional study design was employed in January 2020 among 416 lactating women using systematic sampling techniques. Data was collected using a structured interviewer-administered questionnaire. Bayesian estimation was used on logistic regression to identify the significant predictors of dietary diversity. Convergence of algorithm was assessed by using time series plot, density plot, and autocorrelation plot.

**Result:**

The prevalence of adequate dietary diversity was 23.1%, and the significant predictors of dietary diversity were marital status of mother, education of spouse, occupation of mother and spouse, family size, gravidity, ANC follow up, nutritional education, wealth index, and food security status.

**Conclusion:**

From the result, unmarried, having more family size, multigravidity, poor wealth indexed, and food in secured women were less likely to have adequate dietary diversity, whereas employed women, having ANC follow up and nutrition education, were strongly associated with adequate dietary diversity. Family planning should be given to minimize the impact of large family size of dietary diversity. Any concerned body should give attention to minimize food insecurity of lactating women. Attention should be given for ANC follow-up and nutritional education of mothers by health professional and policy maker.

## 1. Background

A salubrious diet during gravidity and lactation is essential for good alimental health. Maternal malnutrition is reported in different parts of the world, including under nutrition, over nutrition, and micronutrient deficiencies [1–3]. Reproductive age women are vulnerable to nutritional deficiency because of physiological change during the period of pregnancy and lactation. During these periods, the nutrient requirement for women is higher than their counterpart of adult men [4].

The breastfed infant is dependent on mother nutrition, and nutritional needs during lactation are unique and one of the highest demands for good nutrition during a women's life. Virtually, all nutrients have incremented dietary requisites during lactation compared to gravidity. The caloric expenditure of nine months of lactation can exceed that of pregnancy by 98%. Similarly, requirements of most vitamins and minerals increase to varying degrees. Some requirements only increase marginally, such as zinc or potassium, while others increase up to 69% in the case of vitamin A [5, 6].

Good nutrient intake fortifies the stamina, patience, and aplomb that nursing an infant demand. Availing women achieve opportune alimental status to optimize breastfeeding is consequential and requires consideration of energy and nutrient needs. Nutrient requisites are considerably ascended during lactation than in any other stage of a woman's reproductive life. Women who are breastfeeding should increase their energy and nutrient intakes to levels above those of nonpregnant and nonlactating women, since breast milk has to supply an adequate amount of all the nutrients for an infant's needs for growth and development [3, 6–8].

Dietary diversity is defined as the number of individual food items or food groups consumed over a given period of time [2, 9–11]. DD is an essential element of diet quality, and consuming a variety of foods across and within food groups, and across different varieties of specific foods is associated with adequate intake of essential nutrients and other important nonnutrient factors. It is based on the premise that consuming a wide variety of foods will ensure an adequate intake of essential nutrients and, in turn, will lead to better diet quality and optimal health outcomes [12].

Different studies in different parts of the world show that there is a strong association between maternal and child nutritional status with dietary diversity score of lactating mothers [13–16]. Like other developing countries in our country Ethiopia, there is also an evidence of the presences of good nutritional status of children of mothers with high dietary diversity and household and individual food security [4, 14].

Different studies were conducted on the prevalence of adequate dietary diversity and its significant factors. A cross-sectional community-based survey conducted in Southeast Indonesia shows that maternal education and wealth index were associated in the prevalence of high dietary diversity among lactating mothers [17]. The study carried out in West Africa prevails that maternal age, maternal education (secondary+), occupation, literacy, the three empowerment indicators, antenatal attendance, wealth index, and place of residence were determinants of mean dietary diversity score of lactating mothers [15]. Based on the study in Malawi, only 28.1% of lactating mothers meet the mean DDS from nine food groups. As the result of this study, food insecurity was the determinant factor for the prevalence of lower DDS for lactating mothers [18]. A cross-sectional study was done in Kenya which tells that factors that contribute for the prevalence of high dietary diversity in the study were gender, education level of women, age, and family size [19]. The study conducted in Tigray and Southern nations and nationalities, Ethiopia shows that average monthly income, home gardening, source of drinking water, educational status, living in rural area, and food insecurity were significant predictors of the prevalence of low dietary diversity score of lactating mothers [20, 21]. The study survey conducted in the Jimma zone, Southwest Ethiopia, shows that the prevalence of low DDS was 53.46% in three districts. This study revealed that place of residence, maternal age, educational status, and socioeconomic status were strongly associated with low prevalence of DDS in lactating mothers [22]. Another study in Debre tabor, North West Ethiopia, shows that around 75% of the study participant had good dietary diversity during their lactation period, and the prevalence of low DDS was 25.9%. Based on this study, number of live births, the current age of the respondent's, maternal current feeding/meal frequency, and respondent's occupation were factors which associate with the prevalence of low dietary diversity in that study [23]. A cross-sectional study was conducted in South Gondar, Ethiopia, to examine the dietary diversity and associated factors among rural households. The study shows that only 16.2% had high dietary diversity and about 83.8% of participants had inadequate household dietary diversity. The significant predictors of household dietary diversity were radio, mobile phone, bank account, food exchange, and ownership of animals [4]. A community-based cross-sectional survey was conducted in west Gojjam, Ethiopia. This study shows that more than three fourth of the study participants take monotonous food, and the prevalence of high and low dietary diversity score was 10.2% and 53%, respectively [24].

Based on WHO cause of malnutrition framework, dietary diversity (food intake) is one of the immediate causes and DD is a main indicator of diet quality and nutritional outcomes [8].

According to EDHS 2016 and different articles, diversity score among lactating mothers and children is low in Ethiopia [4, 23–27]. In EDHS 2016, the highest prevalence of stunting in under children is recorded in Amhara region which is 46%. From eleven zones of the Amhara region, west Gojjam zone is the most productive and surplus production zone. Despite this high agricultural productivity and wealth index of west Gojjam zone, the prevalence of dietary diversity score was low in this area [26]. Poor dietary diversity causes different nutritional problems, and wasting and underweight were more prevalent in mothers and children who have low dietary diversity score [28]. Lactating mothers who have low dietary diversity score contribute to an increased risk of stunting, wasting, and underweight to their breastfeed child [29].

Ethiopia has been implementing various policies and programs to overcome nutritional problems for the last two decades. Among these, the health extension program was the most popular and effective program that mobilizes the community especially in rural parts of the country. In the beginning of 2018, the Ethiopian government gives a special attention to nutrition and launch a food and nutrition policy [30–32].

Despite these efforts done to overcome nutritional problems, maternal nutrition status was not well improved. Previous literature on the prevalence of adequate dietary diversity has focused basically on qualitative approach. Moreover, the statistical model they employed was more of qualitative and classical approach which cannot show the significant predictors of dietary diversity. Hence, this study tries to assess the prevalence of adequate dietary diversity and to identify the significant predictors of adequate dietary diversity of lactating mother in the study area using Bayesian estimation on logistic regression model.

## 2. Methods

### 2.1. Study Area and Study Design

The study was conducted at Finote Selam District, which is a capital town of west Gojjam Zone, Amhara Region, Ethiopia. Finote Selam means peaceful voyage, first named by Emperor Hailesilassie II. An institution-based cross-sectional study design was employed in which the study population consisted of all Lactating mothers who have postnatal and EPI follow up on government health institutions of study area.

### 2.2. Inclusion and Exclusion Criteria

#### 2.2.1. Inclusion Criteria

All lactating mothers who are on PNC and EPI follow-up during the data collection period were included in the study.

#### 2.2.2. Exclusion Criteria

Lactating mothers who were seriously ill and unable to talk and mothers who were not willing to participate were excluded from the study participation.

### 2.3. Sampling Producer and Sample Size Determination

Systematic sampling technique was used to select a representative sample from target population. Each second mother visiting the PNC and EPI clinic was interviewed after asking her willing and taking informed consent. Sample size is the important point of any research, and the required sample size was determined using single population proportion formula. In this research, the proportion of dietary diversity (56.4%) was taken from previous studies conducted in Aksum, Tigray, Northern Ethiopia, with a 5% margin of error and a 5% of significance level. Based on these assumptions, considering a design effect of 2 since complex sampling was used and adding 10% nonresponse rate, the total sample size was 416.

### 2.4. Study Variables and Data Collection Methods

The response variable of this study was dietary diversity adequacy status. The covariate variables of dietary diversity were taken from related literatures. These variables were age, religion, marital status, gravidity, family size, food security, ANC follow-up, place of residence, occupation of mother, occupation of spouse, educational status of the mother, educational status of the spouse, and received nutrition education ever.

An interviewer-administered questionnaire was used to collect data from respondents. First, the English version of the questionnaire was prepared and translated into Amharic and then back to English by language translators in order to check for consistency. Data on sociodemographic factors was collected by interviewing each of the second lactating mothers visiting the health institution after taking informed consent. To collect dietary history, a 24-hour dietary recall method of dietary history method of nutrition assessment was used to obtain food group consumption information from mothers. The respondents were asked to recall all foods eaten and beverages taken in the previous twenty-four hours prior to the interview date. Dietary diversity scores for the lactating mother were estimated using information collected from the 24-hour dietary recall. For data collection and supervision, there were two clinical nurses and one health officer, respectively.

### 2.5. Bayesian Binary Logistic Regression Model

Binary logistic regression model is used to explain the probability of a binary response variable as function of some covariates [33]. Bayesian logistic regression procedure is used to make inference for the parameters of a logistic regression model. Bayesian statistical methods are becoming ever more popular in applied and fundamental research [34]. This estimation allows a detailed inference from parameters for any arbitrary sample size [35]. Based on the previous study, Bayesian estimation is more accurate than the maximum likelihood estimation even under noninformative prior [36], because this estimation also allows for probabilistic interpretations to the parameters. In general, various studies conclude that Bayesian logistic regression performs better in posterior parameter estimation. There is significant bias of maximum likelihood estimation in small samples, and this weakness can be opposed by using Bayesian logistic regression as an alternative method. Since Bayesian approach is flexible, it does not need to conform to challenging assumptions as proposed in the maximum likelihood method. The Bayesian framework is the mixture of the likelihood function and the prior distribution to develop the posterior distribution [37].

#### 2.5.1. The Likelihood Function

Thus, the response variable *y*_*i*_ follows a Bernoulli distribution with probability *π*. The likelihood function is the probability density function of the data which is seen as a function of the parameter treating the observed data as fixed quantities. For a given sample size *n*, the likelihood function is given as [37]:

(1) $$\begin{array}{c} L\left(\right)=\overset{n}{\underset{i=1}{\prod }}\left[{\left(\frac{e^{\beta_{0}+\beta_{1}X_{i1}+\cdots +\beta_{k}X_{ik}}}{1+e^{\beta_{0}+\beta_{1}X_{i1}+\cdots +\beta_{k}X_{ik}}}\right)}^{y_{i}}{\left(1-\frac{e^{\beta_{0}+\beta_{1}X_{i1}+\cdots +\beta_{k}X_{ik}}}{1+e^{\beta_{0}+\beta_{1}X_{i1}+\cdots +\beta_{k}X_{ik}}}\right)}^{1-y_{i}}\right]. \end{array}$$

#### 2.5.2. Prior Distribution

Prior information is the special feature of Bayesian estimation. In some cases, we may not be in possession of enough prior information to aid in drawing posterior inferences. From a Bayesian point of view, this lack of information is still important to consider and incorporate into our statistical specifications [34]. The most common priors for logistic regression parameters are normal, and the prior distribution of these parameters is given by:

(2) $$\begin{array}{c} P\left(\beta_{j}\right)=\frac{1}{\sqrt{2\pi \sigma_{j}^{2}}}\;\exp\left\{\frac{-1}{2}{\left(\frac{\beta_{j}-\mu_{j}}{\sigma_{j}}\right)}^{2}\right\}. \end{array}$$

The most common choice for prior mean *μj* is 0 for all the coefficients and large enough prior variance *σ*_*j*_^2^ to be considered as noninformative [35].

#### 2.5.3. Posterior Distribution

Bayesian estimation can be done from the posterior distribution, which is derived by multiplying the prior distribution of all parameters and the likelihood function of the data. Then, the posterior distribution is given as follows.

(3) $$\begin{array}{c} \pi \left(\right)=\overset{n}{\underset{i=1}{\prod }}\left[{\left(\frac{e^{\beta_{0}+\beta_{1}X_{i1}+\cdots +\beta_{k}X_{ik}}}{1+e^{\beta_{0}+\beta_{1}X_{i1}+\cdots +\beta_{k}X_{ik}}}\right)}^{y_{i}}{\left(1-\frac{e^{\beta_{0}+\beta_{1}X_{i1}+\cdots +\beta_{k}X_{ik}}}{1+e^{\beta_{0}+\beta_{1}X_{i1}+\cdots +\beta_{k}X_{ik}}}\right)}^{1-y_{i}}\right], \end{array}$$

(4) $$\begin{array}{c} {}*\overset{k}{\underset{j=1}{\prod }}\frac{1}{\sqrt{2\pi \sigma_{j}^{2}}}\;\exp\left\{\frac{-1}{2}{\left(\frac{\beta_{j}-\mu_{j}}{\sigma_{j}}\right)}^{2}\right\}. \end{array}$$

Markov Chain Monte Carlo (MCMC) methods are used to make inference for Bayesian logistic regression models to obtain the posterior distribution of estimation based on a prior distribution and the likelihood function [38]. This method becomes a popular and useful method in Bayesian inference to get information from posterior distributions. The strength of MCMC is that it can be used to draw samples from distributions even when all that is known about the distribution is how to calculate the density for different samples [39]. MCMC sampling method is one very useful class of simulation techniques, and it can simulate a series of dependent random draws from models that are often quite complex.

One popular MCMC method for constructing a Markov chain for a target density is Gibbs sampling. Sampling from the multivariate posterior distribution is not feasible but sampling from the conditional distributions of each parameter is possible; in such cases, Gibbs sampling has been found to be quite applicable. To create the Markov chain, Gibbs sampling uses a set of full conditional distributions associated with the target distribution. Gibbs sampling allows us to use the joint densities that have all other parameters set at their current values [40]. The Gibbs sampler is a special case of the Metropolis-Hastings algorithm using the ordered subupdates. All proposed updates are accepted (there is no accept-reject step). MCMC is (currently) the most general technique for obtaining samples from any posterior density [41]. The Gibbs sampler was implemented easily through WinBUGS software to solve approximates the properties of the marginal posterior distributions for each parameter.

## 3. Results

### 3.1. Sociodemographic Characteristics of the Respondents

The data were analyzed using SPSS 20 for descriptive statistics and Chi-square test, winBUGS Software for Bayesian analysis. In this study, total samples of 416 lactating mothers were considered. Majority of the study respondent were aged less than 30, and most (82.9%) of mothers were Orthodox religion followers. More than half (52.4%) of the respondent were unmarried, and most (61.3%) of mothers were housewives followed by 27.9% employees and 10.8% merchants. Out of the total samples, most of the mothers (68%) and their spouses (64.7%) had secondary and above educational status. The residence of most mothers was urban, and 184 (44.2%) of mothers had a family size of between three and five (Table 1).

Nearly, four-fifth of participants have ANC follow-up and more than half (56.3%) of mothers get nutrition education during ANC follow-up. Most (59.1%) of the respondents were multigirada mothers, and 62% of mothers were poor. Most of the respondent mother were food insecure (62.7%), and the remaining 37.3% of them were food secure (Table 2). Approximately, one-third (23.1%) of the study mothers have adequate dietary diversity, and the rest 76.9% of the respondent have inadequate dietary diversity (Table 3).

Pearson Chi-square test of association was used to identify the rough association between dependent variable and independent variables. This test is the pretest for Bayesian logistic regression analysis. Based on Chi-square test, dietary diversity adequacy was associated with marital status of mother, education of mother and their spouse, occupation of mother and spouse, family size (Table 1), gravidity, ANC follow-up, nutritional education, wealth index, and food security status (Table 2) at 5% level of significance.

### 3.2. Bayesian Logistic Regression Analysis Result

The main objective of this study was to assess the prevalence and significant predictors of adequate dietary diversity among lactating mothers. Bayesian estimation in binary logistic regression analysis was used to identify the significant predictors of adequate dietary diversity. Variables that have *P* < 0.25 on the chi-square test were entered into the Bayesian binary logistic regression model. The Gibbs sampler algorithm was employed on WinBUGS software with 20000 iterations in three different chains, and 57000 samples were getting from the posterior distribution. Assessment of convergence was done using time series plots, density plots, and autocorrelation plots; and based on the plots, the convergence of the algorithm was attained (see supplementary materials (available here)). Accuracy of posterior estimates was assessed based on the Markov Chain (MC) error. An estimate was considered accurate if the MC error is within 5% of the standard deviations. Based on Bayesian estimation, the significant predictors of adequate dietary diversity were marital status of mother, education of spouse, occupation of mother and their spouse, family size, gravidity, ANC follow-up, nutritional education during ANC follow-up, wealth index, and food security status of the respondent (Table 4).

## 4. Discussion

The study has assessed the prevalence of dietary diversity and associated factors among lactating mother visiting governmental health institution in Finote Selam District. Dietary diversity was a proxy indicator for diet quality and nutritional status of both the mother and her child. Children whose mothers have good nutritional status have improved nutrition and health status [14, 16, 42].

Approximately, one-third (23.1%) of the study mothers have adequate dietary diversity, and the rest 76.9% of the respondent have inadequate dietary diversity (Table 3). This result is lower than a research done in Akusum Tigray and Angecha southern nation nationalities region [20, 21]. This difference may be because of seasonal variation in which the data was collected and nutritional knowledge of the community; in addition, food security may be also the causes for this difference. This result is also lower than the finding of study conducted in three countries; Vietnam, Bangladesh, and Ethiopia [43]; in this study, dietary diversity was low in nearly three-fourths of lactating mothers in Ethiopia. The finding on adequate dietary diversity in this study is higher than a survey conducted by [24], Amhara region. In this study, the prevalence of adequate dietary diversity was 10.2%. This may be because of data collection time difference. The survey was conducted on August 14-28, in this time, most of the community was on his farm land, and it is the most difficult time for them. Shortage of food in his time was obvious. An institution-based cross-sectional study conducted in Debre tabor referral hospital results in higher prevalence of good dietary diversity (74.1%) than this finding [23]. This data was collected on September to February, and most crops were cultivated in this season, so this may be the cause of variation between the results. The other reason for the variation may be due to the respondent's occupational status. Most of the respondent was housewife in the current study, but in the previous study, they were government employee and merchants. This can cause economic and knowledge difference about DD.

Marital status of the mother was a significant predictor for adequate dietary diversity. Based on this study, unmarried women were less likely to have adequate DD than married women, and this result is consistent with the study of [44]. Education of spouse was positively and significantly associated with adequate dietary diversity of lactating mothers. The spouse of the mother who had secondary+ education was more likely to have adequate DD than the counterparts. This result was in line with the same research done in Debre tabor and Oromia [23, 44]. From this study, both occupation of mother and their spouse were significantly associated with DD (at least one category). Employee women were more likely to be adequate dietary diversity than housewife women and women who had merchant souse also more likely to be dietary diversity than who had labor souse. This finding is supported by a research done in Debre tabor and Tigray [21, 23]. Family size of women was the significant factor for dietary diversity, and it had a negative effect on adequate DD. Women who have less family size were more likely to have adequate dietary diversity than women who have more family size, and this result is similar with other studies by [19].

Based on the finding, gravidity and ANC follow-up were significant predictors of dietary diversity. Moreover, women who had multi gravid were less likely to have adequate dietary diversity than counterpart, and women who had ANC follow-up were more likely to have adequate dietary diversity. There is also strong association between nutrition education during ANC follow-up and dietary diversity of lactating mother. Women who got nutrition education during ANC follow-up were more likely to have adequate DD. As far as we know, there is no article done with nutrition education during ANC follow-up and DD. But finding prevails that educational status was significantly associated with maternal dietary diversity [19, 21, 44, 45]. Since education can increase the knowledge of mother about nutrition, this may be in line with this finding. Wealth index was strongly associated with DD of lactating mother. The probability of having adequate dietary diversity in rich mothers was more likely than mothers who are poor. This result is the same with other studies done by [7, 20–22], in Tigray, SNNP Region, Jimma Oromia region, Kenya Nairobi, and a survey done by USAID in different countries of the world. Last, food security was another strong significant factor for the dietary diversity of lactating mother. The likelihood of being adequately dietary diversified was more likely in food secure mothers than food insecure mothers. This result was the same with other studies conducted by the following researchers [18, 21, 43, 46, 47]. Most research findings confirm that food security and DD were significantly associated in different regions.

## 5. Conclusion

The objective of this study was to assess the prevalence and significant factors of dietary diversity of lactating mother. Among 416 samples, the prevalence of adequate dietary diversity among lactating mother was 23.1%, and this result was supported by other studies in different countries and regions of the country. From Bayesian logistic regression analysis, variables like marital status of mother, education of spouse, occupation of mother and spouse, family size, gravidity, ANC follow-up, nutrition education, wealth index, and food security status were significant predictors of dietary diversity of lactating mother. Moreover, unmarried women, women having more family size and multi gravidity, poor wealth indexed, and food in secured women were less likely to have adequate dietary diversity, whereas employed women, women having ANC follow-up, and nutrition education were strongly associated with adequate dietary diversity. Based on the result, family planning should be given to reduce family size to minimize the impact of large family size of dietary diversity. Different mechanisms of getting food should be considered by any concerned body to minimize food insecurity of lactating women. Occupation for women should be emphasized to enable them to gain greater access and control over financial and knowledge resources to improve their lives and diets. Attention should be given for ANC follow-up and nutritional education of mothers by health professional and policy maker.

## Figures and Tables

**Table 1:** Sociodemographic characteristics of lactating mother in Finote Selam District.

Variable	Category	Frequency (%)	Chi-square Pearson value (sig.)
Age	<30	270 (64.9)	0.256 (0.880)
	30-40	116 (27.9)	
	>40	30 (7.2)	
Religion	Orthodox	345 (82.9)	0.654 (0.419)
	Muslim	71 (17.1)	
Marital status	Married	198 (47.6)	92.640 (0.000)
	Unmarried	218 (52.4)	
Educational status of mother	Illiterate	64 (15.4)	18.619 (0.000)
	Primary	69 (16.6)	
	Second and above	283 (68)	
Educational status of spouse	Illiterate	78 (18.8)	39.835 (0.000)
	Primary	69 (16.6)	
	Second and above	269 (64.7)	
Occupation of mother	Housewife	255 (61.3)	16.960 (0.000)
	Employee	116 (27.9)	
	Merchant	45 (10.8)	
Occupation of spouse	Daily laborer	126 (30.3)	27.920 (0.000)
	Employee	167 (40.1)	
	Merchant	123 (29.6)	
Residence	Urban	399 (95.9)	1.278 (0.258)
	Rural	17 (4.1)	
Family size	<3	147 (35.3)	79.589 (0.000)
	3-5	184 (44.2)	
	≥6	85 (20.4)	

**Table 2:** Obstetrics and economic-related characteristics of the respondents.

Variable	Category	Frequency (%)	Chi-square Pearson value (sig.)
Gravidity	Primi gravid	170 (40.9)	63.902 (0.000)
	Multi gravid	246 (59.1)	
ANC follow-up	No	87 (20.9)	11.941 (0.001)
	Yes	329 (79.1)	
Nutrition education on ANC	Yes	234 (56.3)	97.067 (0.000)
	No	182 (43.8)	
Wealth index	Poor	258 (62.0)	54.399 (0.000)
	Medium	47 (11.3)	
	Rich	111 (26.7)	
Food security	Food insecure	261 (62.7)	118.510 (0.000)
	Food secure	155 (37.3)	

**Table 3:** Dietary diversity of lactating mothers.

		Frequency	Percentage
Dietary diversity	Inadequate	320	76.9
	Adequate	96	23.1

**Table 4:** Results of Bayesian analysis of dietary diversity.

Variable	Category	Mean	Sd	MC error	95% credible interval	
					Lower	Upper
Constant		-15.37^∗^	3.152	0.1103	-22.01	-9.813
Marital status	Unmarried	-8.041^∗^	1.614	0.03359	-11.47	-5.179
Education of mother	Primary	-1.737	2.176	0.03715	-6.037	2.513
	Secondary+	0.0457	1.735	0.04161	-3.192	3.616
Education of spouses	Primary	0.6225	2.45	0.03675	-4.271	5.334
	Secondary+	3.459^∗^	1.793	0.04499	0.1553	7.18
Occupation of mother	Employee	2.845^∗^	1.034	0.01682	0.8927	4.942
	Merchant	0.8514	1.182	0.01181	-1.47	3.18
Occupation of spouses	Employee	1.894	1.509	0.03468	-1.002	4.941
	Merchant	6.642^∗^	1.912	0.05341	3.159	10.62
Family size	3-5	3.737^∗^	1.246	0.0224	1.457	6.338
	≥6	6.639^∗^	1.662	0.03137	3.66	10.18
Gravidity	Multi gravid	-6.562^∗^	1.35	0.02691	-9.428	-4.138
ANC follow-up	Yes	2.404^∗^	1.144	0.02281	0.2505	4.73
Nutrition of education	Yes	2.553^∗^	0.912	0.01428	0.8651	4.443
Wealth index	Medium	3.898^∗^	1.343	0.0149	1.359	6.628
	Rich	4.452^∗^	1.171	0.02255	2.295	6.881
Food security	Yes	5.769^∗^	1.157	0.02543	3.714	8.259

^∗^Significant at 5% level of significance, credible interval = 95%.

## Data Availability

The primary data set collected from households and analyzed during the current study is available from the corresponding author

## Abbreviations

ANC: Antenatal care
CSA: Central statistical agency
DD: Dietary diversity
DDS: Dietary diversity score
EDHS: Ethiopian Health and Demographic Survey
EPI: Expanded program on immunization
HFIAS: Household food insecurity access scale
MDDS: Mean dietary diversity score
NNS: National Nutrition Strategy
NNP: National Nutrition Program
PNC: Postnatal care
UNICEF: United Nation International Children's Economic Fund
WHO: World Health Organization.

## Supplementary Materials

Supplementary Materials Different plots to check the convergence of algorithm. Time series plots of significant predictors. Gelman Plots of the model. Density Plots. Autocorrelation plots.

## References

[1] Breda J., Robertson A. (2016). Good Maternal Nutrition: The Best Start in Life.

[2] FAO F (2016). *Minimum Dietary Diversity for Women: A Guide for Measurement*.

[3] Kominiarek M. A., Rajan P. (2016). Nutrition recommendations in pregnancy and lactation. *Medical Clinics*.

[4] Girma N., Degnet T. (2015). Dietary diversity and associated factors among rural households in South Gondar Zone, Northwest Ethiopia. *Feed the Future*.

[5] Pratt CSaN (2019). *Nutrition during Lactation: What Do Mom and Baby Need?*.

[6] Picciano M. F. (2003). Pregnancy and lactation: physiological adjustments, nutritional requirements and the role of dietary supplements. *The Journal of Nutrition*.

[7] Ongosi A. N. (2010). *Nutrient Intake and Nutrition Knowledge of Lactating Women (0-6) Months Postpartum in a Low Socio-Economic Area in Nairobi*.

[8] Organization WH (2013). *Essential Nutrition Actions: Improving Maternal, Newborn, Infant and Young Child Health and Nutrition*.

[9] Kennedy G., Ballard T., Dop M. C., Division FaAOotUNNaCP (2010). Guidelines for measuring household and individual dietary diversity. *Nutrition and Consumer Protection Division*.

[10] Nithya D., Bhavani R. (2018). Dietary diversity and its relationship with nutritional status among adolescents and adults in rural India. *Journal of Biosocial Science*.

[11] Waswa L. M. (2015). *Improving Dietary Diversity and Nutritional Health of Women and Children under Two Years through Increased Utilization of Local Agrobiodiversity and Enhanced Nutrition Knowledge in Kenya*.

[12] de Oliveira Otto M. C., Anderson C. A., Dearborn J. L. (2018). Dietary diversity: implications for obesity prevention in adult populations: a science advisory from the American Heart Association. *Circulation*.

[13] Arimond M., Ruel M. T. (2004). Dietary diversity is associated with child nutritional status: evidence from 11 demographic and health surveys. *The Journal of Nutrition*.

[14] Melaku Y. A., Gill T. K., Taylor A. W., Adams R., Shi Z., Worku A. (2018). Associations of childhood, maternal and household dietary patterns with childhood stunting in Ethiopia: proposing an alternative and plausible dietary analysis method to dietary diversity scores. *Nutrition Journal*.

[15] Amugsi D. A., Mittelmark M. B., Oduro A. (2015). Association between maternal and child dietary diversity: an analysis of the Ghana demographic and health survey. *PLoS One*.

[16] Ocampo-Guirindola M. L., Garcia-Malabad C. J., Valdeabella-Maniego M. L. M., Punzalan S. L. M. (2016). Association between dietary diversity score and nutritional status of Filipino children aged 6-23 months. *The Philippine Journal of Science*.

[17] Rahmannia S., Diana A., Luftimas D. E. (2019). Poor dietary diversity and low adequacy of micronutrient intakes among rural Indonesian lactating women from Sumedang district, West Java. *PLoS One.*.

[18] Kang Y., Hurley K. M., Ruel-Bergeron J. (2019). Household food insecurity is associated with low dietary diversity among pregnant and lactating women in rural Malawi. *Public health nutrition*.

[19] Gitagia M. W., Ramkat R. C., Mituki D. M., Termote C., Covic N., Cheserek M. J. (2019). Determinants of dietary diversity among women of reproductive age in two different agro-ecological zones of Rongai Sub-County, Nakuru, Kenya. *Food & Nutrition Research*.

[20] Weldehaweria N. B., Misgina K. H., Weldu M. G. (2016). Dietary diversity and related factors among lactating women visiting public health facilities in Aksum town, Tigray, Northern Ethiopia. *BioMed Central nutrition*.

[21] Boke M. M., Geremew A. B. (2018). Low dietary diversity and associated factors among lactating mothers in Angecha districts, southern Ethiopia: community based cross-sectional study. *BMC research notes*.

[22] Sirawdink F. Forsidoac TBaOH Fighting hidden hunger: Diversity, Composition and Nutrient Adequacy of Diets of Lactating Mothers in Jimma Zone, Southwest Ethiopia.

[23] Engidaw M. T., Gebremariam A. D., Tiruneh S. A., Asnakew D. T., Abate B. A. (2018). Dietary diversity and associated factors among lactating mothers in Debre Tabor General Hospital, Northcentral Ethiopia. *International Journal*.

[24] Ahmen A., EPH I. (2014). *Assessment of Dietary Diversity among Pregnant and Lactating Women and 6 to 23 Months Age Children, in Rural Areas of Western Gojjam, Amhara Region*.

[25] Bogale S. (2014). *Graduation with Resilience to Achieve Sustainable Development Nutrition Baseline Report Addis Ababa Ethiopia*.

[26] Mekuria G., Wubneh Y., Tewabe T. (2017). Household dietary diversity and associated factors among residents of Finote Selam town, north west Ethiopia: a cross sectional study. *BMC Nutrition*.

[27] Zerfu T. (2017). Ethiopian Demographic and Health Survey.

[28] Sealey-Potts C., Potts A. (2014). An assessment of dietary diversity and nutritional status of preschool children. *Austin Journal of Nutrition and Food Sciences*.

[29] Huang M., Sudfeld C., Ismail A. (2018). Maternal dietary diversity and growth of children under 24 months of age in rural Dodoma, Tanzania. *Food and nutrition bulletin*.

[30] Mo H., Ethiopia GoFRO (2013). *National Nutrition Programme One*.

[31] Fdro E., Mo H. (2016). *National Nutrition Program*.

[32] Ethiopia F. R. O., Mo E. (2017). *Emergency School Feeding Program*.

[33] Agresti A. (2007). *Categorical Data Analysis*.

[34] van de Schoot R., Kaplan D., Denissen J., Asendorpf J. B., Neyer F. J., van Aken M. A. G. (2014). A gentle introduction to Bayesian analysis: applications to developmental research. *Child Development*.

[35] Anjullo B. B., Haile T. T. (2018). A Bayesian binary logistic regression approach in identifying factors associated with exclusive breastfeeding practices at Arba Minch Town, South Ethiopia. *Advances in Research*.

[36] Rashwan N. I., Eldereny M. (2012). The comparison between results of application Bayesian and maximum likelihood approaches on logistic regression model for prostate cancer data. *Applied Mathematical Sciences*.

[37] Chiaka E. S., Adam M. B. (2019). Bayesian analysis via Markov chain Monte Carlo algorithm on logistic regression model. *Global Journal of Pure and Applied Mathematics*.

[38] Souza A. D. P., Migon H. S. (2004). Bayesian binary regression model: an application to in-hospital death after ami prediction. *Pesquisa Operacional*.

[39] van Ravenzwaaij D., Cassey P., Brown S. D. (2018). A simple introduction to Markov chain Monte–Carlo sampling. *Psychonomic bulletin & review*.

[40] Gearhart C. (2018). *Implementation of Gibbs Sampling within Bayesian Inference and Its Applications in Actuarial Science*.

[41] Holmes C. *Markov chain Monte Carlo and applied Bayesian statistics: a short course*.

[42] Negash C., Whiting S. J., Henry C. J., Belachew T., Hailemariam T. G. (2015). Association between maternal and child nutritional status in Hula, Rural Southern Ethiopia: a cross sectional study. *PLoS One*.

[43] Nguyen P. H., Avula R., Ruel M. T. (2013). Maternal and child dietary diversity are associated in Bangladesh, Vietnam, and Ethiopia. *The Journal of nutrition*.

[44] Agize A., Jara D., Dejenu G. (2017). Level of knowledge and practice of mothers on minimum dietary diversity practices and associated factors for 6–23-month-old children in Adea Woreda, Oromia, Ethiopia. *BioMed research international*.

[45] Henjum S., Torheim L. E., Thorne-Lyman A. L. (2015). Low dietary diversity and micronutrient adequacy among lactating women in a peri-urban area of Nepal. *Public health nutrition*.

[46] Adubra L., Savy M., Fortin S. (2019). The minimum dietary diversity for women of reproductive age (MDD-W) indicator is related to household food insecurity and farm production diversity: evidence from rural Mali. *Current Developments in Nutrition*.

[47] Belachew T., Lindstrom D., Gebremariam A. (2013). Food insecurity, food based coping strategies and suboptimal dietary practices of adolescents in Jimma zone Southwest Ethiopia. *PLoS One*.

