# Supplementary material for: Bayesian Analysis of Dietary Diversity among Lactating Mothers in Finote Selam District, Northwest Ethiopia: A Cross-Sectional Study
Source: Biomed Res Int. 2021 Aug 29;2021:9604394. doi: 10.1155/2021/9604394 (PMC8421177; doi:10.1155/2021/9604394)

**Supplementary Materials**

**Different plots to check the convergence of algorithm.**

1. **Time series plots of significant predictors**

1. **Gelman Plots of the model**


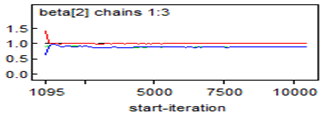

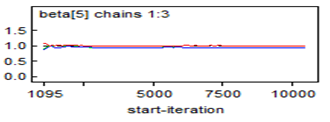


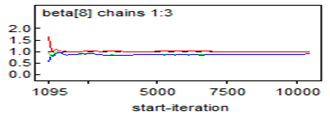

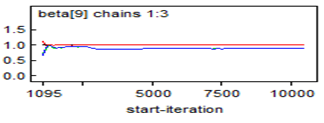


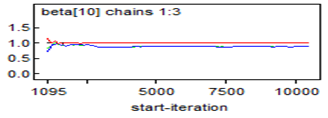

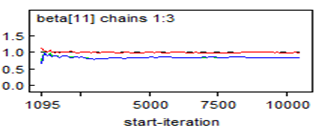


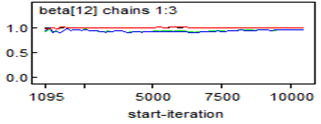

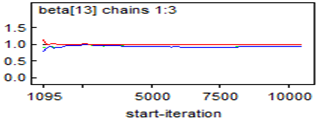


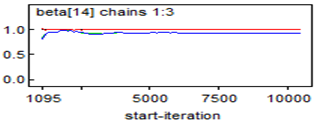

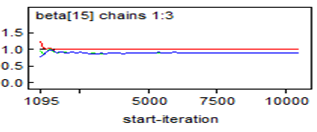


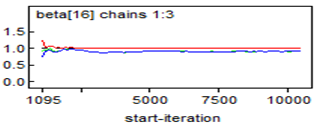

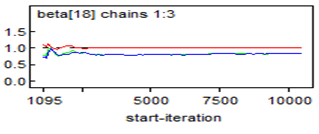


1. **Density Plots**


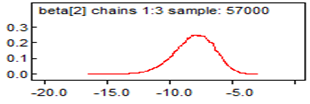

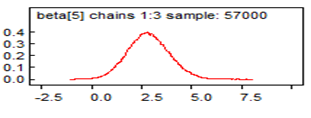


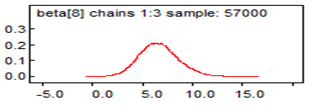

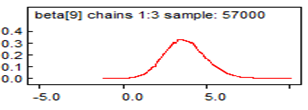


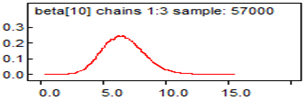

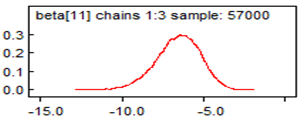


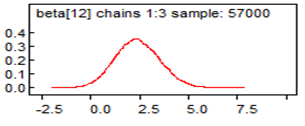

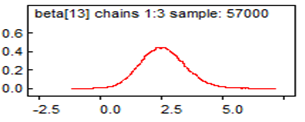


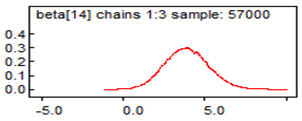

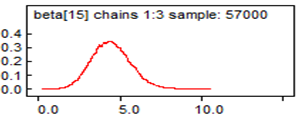


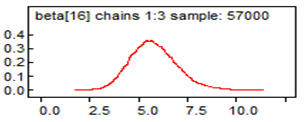

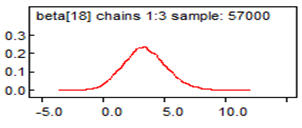


1. **Autocorrelation plots**


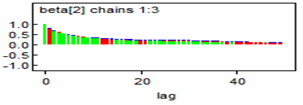

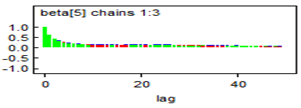


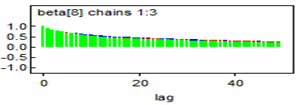

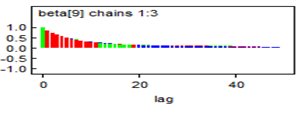


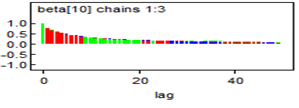

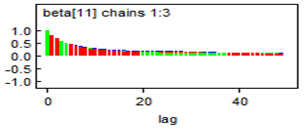


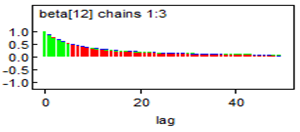

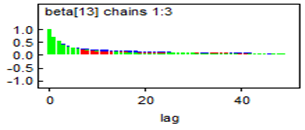


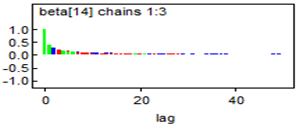

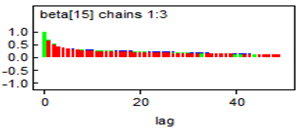


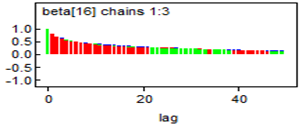

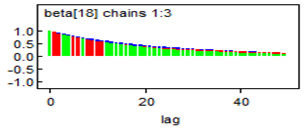

Supplement: Supplementary Materials — Different plots to check the convergence of algorithm. Time series plots of significant predictors. Gelman Plots of the model. Density Plots. Autocorrelation plots. [file 9604394.f1.docx]
